# Detection and continuous monitoring of moisture content in transformer oil using fractal-based capacitive sensor

**DOI:** 10.1016/j.heliyon.2024.e40995

**Published:** 2024-12-09

**Authors:** Muhammad Fahad Naveed, Akasha Amaar, Saad Saleem Khan, Muhammad Omar, Stephen Larkin

**Affiliations:** a Frankfurt University of Applied Sciences, Germany; b Technical University of Dortmund, Germany; c United Arab Emirates University, Al-Ain, United Arab Emirates; d Africa New Energies, UK

**Keywords:** Capacitive sensor, Fractal, IoT, Moisture contamination, Real-time monitoring, Transformer oil

## Abstract

The contaminated transformer oil is one of the major causes of failure in the power system. Detection and continuous monitoring of moisture content in transformer oil is required for the smooth operation of a system. In this paper, a Fractal-based Sinusoidal-shaped Capacitive Sensor (FSCS) is proposed to increase the contact area between capacitor plates and dielectric medium by 17.3%. The proposed design offers minimal resistance to the flow of a mixed medium and can detect moisture content accurately. The variation in transformer oil, water, and mixture due to the temperature is considered to avoid false readings and alarms. The Equivalent Series Resistance (ESR) is also measured along with the equivalent capacitance to increase the accuracy of FSCS. A minimum detection limit of 5PPM is observed for a proposed sensor. The switching mechanism is integrated with the proposed design to make it reconfigurable and change the contact area with the impurity and sensitivity of the sensor. The large contact area and higher sensitivity mode are activated separately in order to avoid false readings. The sensor with a higher fractal stage achieves a sensitivity (fF/5PPM) that is 18.22 times greater than that of the lower fractal stage, making the higher stage more sensitive. As a result of switching, further substages of the fractal stage are formed. Each fractal stage can have multiple substages. The sensitivity of any particular substage increases as its fractal stage increases.

## Highlights

• A new Fractal-based capacitive sensor is proposed to detect the moisture content in transformer oil.
• The substages are used to make the sensor reconfigurable.
• The switching mechanism is introduced to switch between further substages of a Fractal stage.

## Graphical abstract

**Figure:**
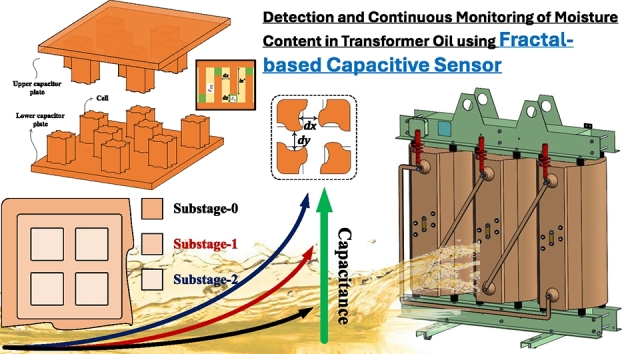

## 1 Introduction

Power transformers are a core component of electrical transmission and distribution networks [1]. The significance of power transformers continues to grow as the demand for electricity surges. The reliable and continued performance of a power transformer is the key to the profitable generation and transmission of electric power.

A transformer comprises of many essential components, including the laminated core, windings, transformer oil, and bushings. Transformer oil, also referred to as insulating oil, serves a pivotal role as a dielectric medium within the transformer, preventing electrical breakdown. It also plays a crucial role in dissipating the heat generated during transformer operation, thus maintaining optimal temperature levels [2].

The failure of a transformer can be attributed to a combination of electrical, mechanical, or thermal factors [3], [4]. These faults can be categorized as internal as well as external. Lightning strikes, overloading, and short circuits are some of the main causes of external faults in transformers. Internal faults in a transformer mostly occur when internal components of the transformer fail to work; insulation deterioration, winding failure, overheating, and contamination of oil are some examples of internal faults in a power transformer. A statistical analysis has been done that determines the main causes of power failure [5]. The results show that the failures due to the insulation constitute about 30%, followed by the winding, leakage, and bushing with 13%, 12%, and 12%, respectively [6], [7].

The presence of different contaminants like metal and dust particles, oxidation, overheating, powder, and moisture can lead to the failure of transformer oil [8], [9]. However, this work is only focused on the detection of moisture content in the transformer oil.

Since water is a threat to the insulation system of a power transformer, determining the water content is of great significance for maintaining stable operation and avoiding any damage to the transformer [10]. A lot of work has been done in this respect involving different techniques and technologies. There are invasive and non-invasive techniques to monitor the health of transformer oil.

It is a challenge to provide both accuracy and minimal cost in a single device. Traditional methods to detect the presence of moisture in transformer oil include the Visual Crackle Test Recovery Voltage Measurement [11], [12], [13] and the Karl Fischer Method [14], [15]. The Visual Crack Method was the simplest and the earliest method known. The oil sample is collected and dropped on a high-temperature plate where the presence of moisture content is observed through bubbles and explosion sounds of water droplets. This measurement method is not quantitative and is the least accurate. Similarly, the Recovery Voltage Method estimates the humidity of the transformer oil and does not require any calibration [11]. The values deduced are highly influenced by temperature and oil conductivity, thus making it less accurate. Also, it does not allow moisture detection of less than 1% [13].

Karl Fischer is a chemical method and is considered accurate and sensitive among other traditional methods. A titrant is added, which reacts with the water in transformer oil and generates the current, which is then measured to detect the moisture content. It consists of mainly two methods: coulometric and volumetric [16]. The detection range of this approach is as low as 1PPM for the coulometric method and 50-100PPM for the volumetric method [17], [18]. Despite being efficient, this method has two main disadvantages: it is highly expensive and only allows offline detection. Also, it is time-consuming, laboratory-based, and uses hazardous chemicals.

Other than the conventional methods discussed above, MEMS technology has also been introduced in different forms to detect the moisture content in transformer oil. Optical sensors are worth mentioning as they are highly accurate [19], [20], [21]. Optical sensors are used to monitor the moisture content in transformer oil by monitoring the change in refractive index. The contaminated transformer oil has a different refractive index as compared to the pure one. This phenomenon is highly accurate but, at the same time, extremely expensive. Dissolved Hydrogen Gas Analysis is another method to monitor the health of transformer oil. A MEMS-based sensor array is proposed in [22], which contains Wheatstone bridge circuitry with two pairs of resistors. The microwave-based sensors are also discussed in the literature for the detection of moisture content. One of many microwave-based sensors is discussed in [23], which operates in the frequency band of 90-190 MHz. It is directly implemented on the pipe surface due to its planar configuration. The sensor is tested for the efficient oil production and refining process. Another method for the detection of moisture is presented in [24], in which a humidity sensor is developed using material-reduced graphene oxide and molybdenum disulfide (rGO: MoS2). It offers a high surface area, a large number of active sites, and excellent mechanical flexibility.

In the literature, several capacitive sensors are also discussed for the same purpose of moisture detection. The interdigitated sensors as capacitive sensors for the sensing of permittivity and conductivity of oils are presented in [25]. The parasitic capacitances are reduced to avoid inaccuracy. A cross-capacitance sensor developed in [26] is used to measure the moisture and 2-Furfuraldehyde in transformer oil. It is developed using bulk brass material and consists of four identical brass electrodes that are separated by very small gaps. The condition of the oil is determined by adding moisture in the range of 0-60PPM. A planar metal-polyimide interdigital transducer (IDT) miniaturized capacitive sensor is developed in [27] for real-time detection of moisture with a sensitivity of 1.60pF per percent of change in relative humidity. Another capacitive moisture sensor made up of thin films of nano-porous alumina dip-coated in between two parallel gold electrodes is shown in [28].

In all of the above-discussed papers, there is no reconfigurability. The proposed sensor can be operated either in sensitive or large contact area mode. Please note that the large contact area mode allows for the increased contact area between the capacitor electrode and the impurity. Both modes are suitable for different situations; for instance, the sensitive mode is activated when the transformer oil is in normal condition. Once there is any abnormality in the capacitance is found, large contact area mode can be used to cross-check the moisture presence and avoid any false alarm.

**Figure 1:**
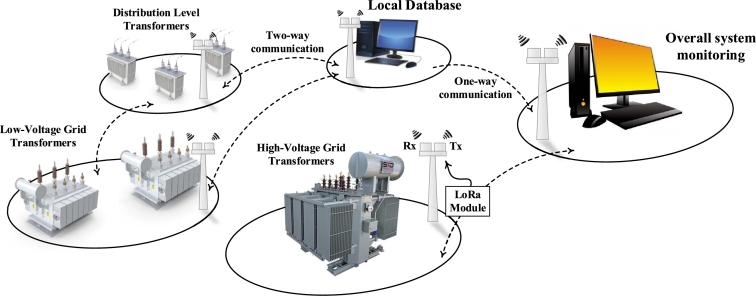
A network containing multiple nodes communicating (one-way or two-way) with each other using a Long-Range (LoRa) module for the continuous monitoring of the transformer oil.

The schematic diagram of the network containing transformers is shown in Fig. 1. An IoT-based device is connected to multiple nodes for online monitoring. The one-way and two-way communication are possible between the nodes for the transfer of data. Local databases are present in between the nodes and the overall system of monitoring. The LoRa module is used along with the FSCS to transfer the data over a long range of distances. Continuous monitoring is important as it can help to identify problems, such as Degradation of the insulation, Leaks, Partial Discharge, and Contamination, beforehand to prevent a lot of problems, including transformer failure and power outages. The parameters used throughout the paper are summarized in Table 1.

**Table 1:** Parameters.

Symbol	Definition	Symbol	Definition
*a*_(*i*)_	Variable 1	*lz*_(*i*)_	Modified length of a cell along the z-axis
*b*_(*i*)_	Variable 2	*lz*_′_	Length of a cell along the z-axis
$Cx_{eq}^{\left[i\right]}$	Equivalent capacitance along the x-axis direction	*lz*	Constant modified length of a coil along the z-axis
$Cy_{eq}^{\left[i\right]}$	Equivalent capacitance along the y-axis direction	*l*_(*i*)_	Length of the wavy side along the x and y-axis
$Cz_{eq}^{\left[i\right]}$	Equivalent capacitance along the z-axis direction	$L_{\left(i\right)}^{\prime }$	A straight length of the ith stage along the x and y-axis
*Cx*_1×1_	The capacitance between two cells along the x-axis	N	Last fractal stage
*Cz*_1×1_	The capacitance between two cells along the z-axis	N.C	Normally close
$C_{eq}^{\left[i\right]}$	Equivalent capacitance of overall design	*n*_(*i*)_	No. of cells along the x or y-axis
$C_{1}^{\left[sub-1\right]}$	The capacitance of one unit in substage 1	*n*^[*sub*−*j*]^	No. of cells along the x or y-axis in substage N
*dx*_(*i*)_	Distance between two cells along the x-axis	*n*^[*sub*−(*j*−1)]^	No. of cells along the x or y-axis in substage (N-1)
*dy*_(*i*)_	Distance between two cells along the y-axis	PPM	Parts Per Million (volume ratio)
*dz*	Distance between cell and plate along the z-axis	*R*_*internal*_	The internal resistance of the function generator
*d*_(*i*)_	Distance between two cells of the ith stage along the x and y-axis	*R*_*series*_	Series resistance
*d*	Constant distance between two cells along the x and y-axis	*ρ*_*m*_	Resistivity of oil and water mixture
*ε*	Relative permittivity	*ρ*_*oil*_	Resistivity of oil
*ε*_0_	The permittivity of free space	*ρ*_*water*_	Resistivity of water
*ε*_*s*_	The permittivity of supporting structure	*r*	Variable 3
*ε*_*m*_	The permittivity of oil and water mixture	T	Temperature
$\varepsilon_{m}^{\left[sub-j\right]}$	The permittivity of the oil and water mixture at the *j*^*th*^ substage	$T_{1}^{\left[sub-1\right]}$	Terminal of one unit in substage 1
*ε*_*oil*_	Permittivity of oil	*u*,*v*	Function dependent upon compound type and behaviour
*ε*_*water*_	Permittivity of water	*V*_(*i*)_	The total volume of a design at the stage
ESR	Equivalent Series Resistance	*V*_*a*_	The voltage across the series resistor and capacitor
*i*	fractal stage	*V*_*b*_	Voltage across capacitive sensor under test
*j*	substage	*V*_*source*_	Input voltage source
$L_{initial}^{\prime }$	Straight length of Stage 0 along the x or y-axis	$V_{R_{series}}$	The voltage across a series resistor
*lx*_(*i*)_	Length of the wavy side along the x-axis	*X*,*Y*	Fraction of oil and water
*ly*_(*i*)_	Length of the wavy side along the y-axis	*ϕ*	Loss angle

In this paper, a novel Fractal-based Sinusoidal-shaped Capacitive Sensor (FSCS) is presented. The detail of the design is discussed in section 2. The FSCS is optimally designed to provide minimal resistance to the flow of transformer oil and increase the contact area by approximately 17.3% at the same time. The main purpose of proposing this design is to be able to reconfigure the sensor to increase either sensitivity or contact area with the impurity. This reconfigurability is achieved by introducing the concept of *i* substages which is explained in detail in section 3. The ESR and Capacitance are measured to detect and accurately quantify the moisture content present in the transformer oil under test. The process of fabrication and experimental setup is described in section 4. The results of simulation and practical are discussed in section 5.

## 2 Design and mathematical modeling

**Figure 2:**
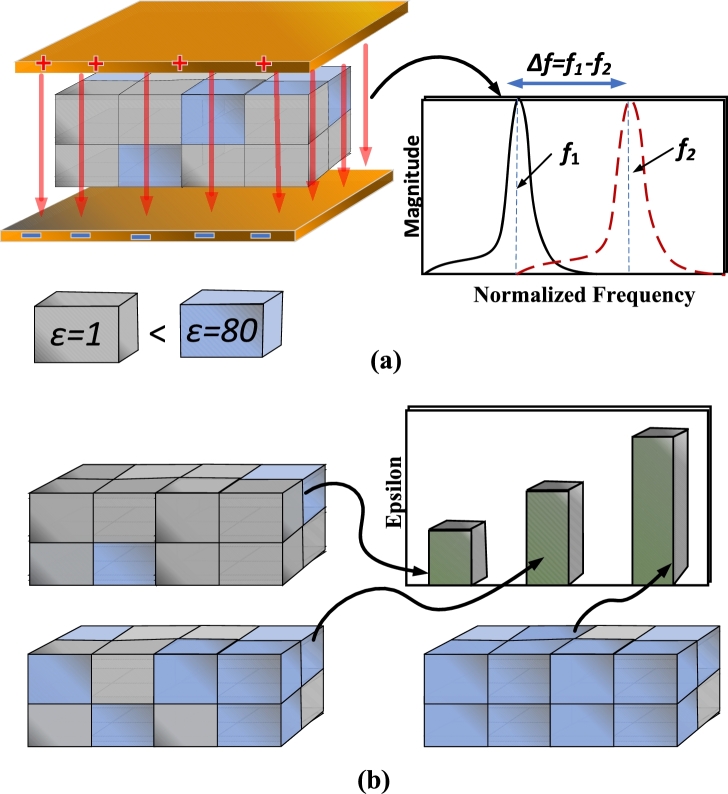
(a) Frequency shift due to the moisture content between the plates of the capacitor (b) Effect of different concentrations of water on the dielectric constant.

The dielectric constant of water is significantly greater than that of oil, imparting a pronounced influence on capacitance. As depicted in Fig. 2 (a), a noticeable trend emerges: in the case of the presence of water droplets between the conductors, there is a frequency shift denoted by Δf. This trend continues to shift with the increasing quantity of water because of an increase in the dielectric constant. In addition, the distribution of water within this context is anything but uniform. As water droplets infiltrate the region between the electrodes of a capacitor, the dielectric constant undergoes a continuous increase, as illustrated in Fig. 2 (b).

**Figure 3:**
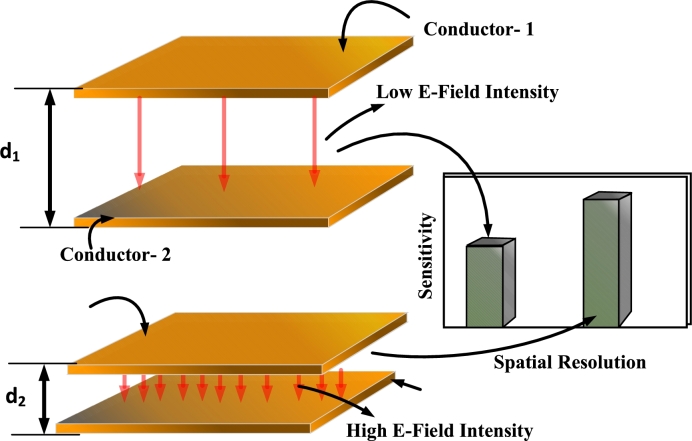
Effect on the sensitivity of the capacitor with the decrease in distance between the plates.

Owing to the non-uniform water distribution, a need arises for sensing across multiple points. Furthermore, a high level of sensitivity is indispensable. Increasing the surface area of the capacitor enhances the capacitance and sensitivity of the capacitor. Similarly, decreasing the distance between the electrodes results in a stronger electric field as shown in Fig. 3. This results in an enhanced spatial resolution, and consequently, the sensitivity of the sensor to variations in the capacitance is increased.

**Figure 4:**
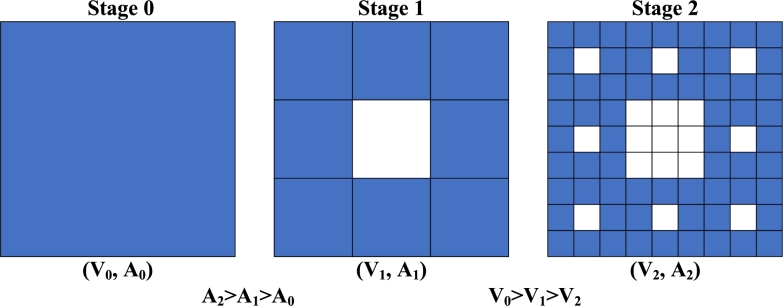
Fractal stages for a square indicating the increase in area and decrease in volume with increasing stages.

These considerations have led to the adoption of a fractal-shaped capacitor. By increasing the number of fractal stages, the area of the conductor expands significantly, resulting in a significant increase in capacitance and sensitivity. Different stages of the fractal are shown in Fig. 4 which shows an increase in area with the increase in the number of stages.

In this section, the impact of fractal stages on the proposed capacitive sensor is discussed. The state-of-the-art sensor is proposed, which includes Sinusoidal-shaped sides, unlike Rectangular-shaped ones. The contact area between transformer oil and capacitor plates is increased to enhance sensitivity. A detailed mathematical modeling of the FSCS is also disclosed.

### 2.1 Fractal stages

**Figure 5:**
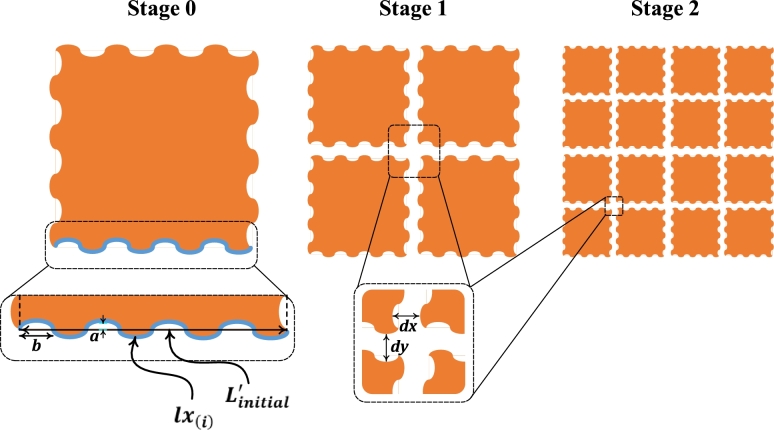
Three fractal stages of a proposed Sinusoidal-shaped Capacitive Sensor design having constant distance between two cells across all Stages.

The presented design shown in Fig. 5 is based on fractal stages due to its unique behaviour of the increase in surface area while the decrease in volume. It is mandatory for a proposed design to be able to remain in contact with the fluid (transformer oil) as much as possible for better sensing. To do so, the surface area of the capacitor plates needs to be increased.

### 2.2 State-of-the-art capacitor design

In this section, a state-of-the-art capacitive sensor design is shown. To better understand the working and design of a sensor, the fractal stage 2 is demonstrated here. The same concepts can be applicable to lower or higher stages.

**Figure 6:**
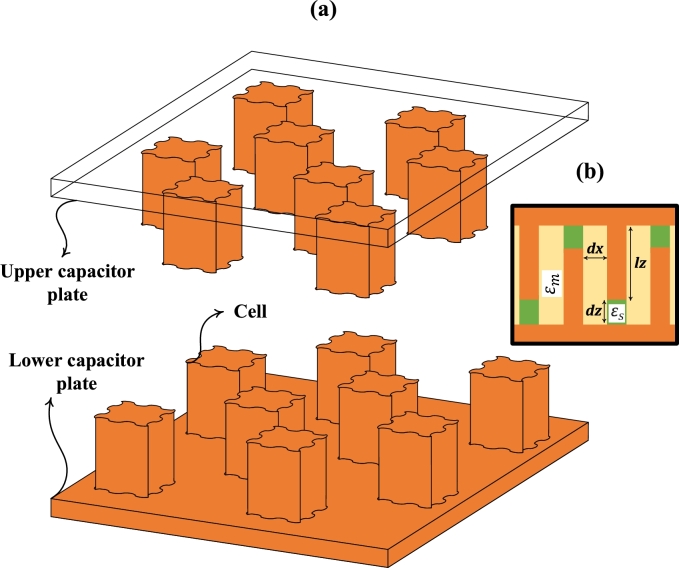
(a) Exploded Isometric View of a Fractal Sinusoidal-shaped Capacitive Sensor (b) Side View of a Capacitive sensor showing transformer oil and supporting structure.

As shown in Fig. 6, there are multiple cells, each having higher or lower potential. In this scenario, cells having higher potential are considered to be attached to the upper capacitor plate, as shown in Fig. 6 (a). At the same time, the lower capacitor plate contains the cells with lower potential. Due to the alternate polarity on nearby cells, multiple parallel capacitances are formed. The collective capacitance of the design is named “equivalent capacitance”.

The supporting structure between the upper and lower plates of the FSCS is made up of FR4 substrate, as shown in Fig. 6 (b). It is used to provide support as well as insulation between two opposite polarity plates. $Cz_{eq}^{\left[i\right]}$ is the equivalent capacitance of multiple capacitors formed between cells and lower or upper plates through FR4.

**Figure 7:**
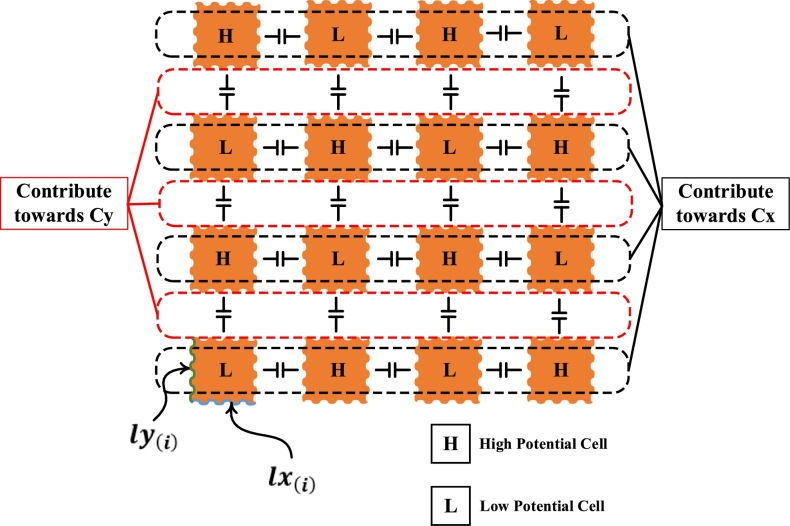
Fractal Stage 2 of a proposed Capacitive Sensor having different polarities on alternate cells with multiple capacitors contributing towards Cx and Cy.

Due to the formation of capacitance in different orientations, it is categorized into $Cx_{eq}^{\left[i\right]}$, $Cy_{eq}^{\left[i\right]}$, and $Cz_{eq}^{\left[i\right]}$. The $Cx_{eq}^{\left[i\right]}$ is the equivalent capacitance of all capacitors formed in the direction of the x-axis, while $Cy_{eq}^{\left[i\right]}$ is due to the y-axis capacitors as shown in Fig. 7.

In the presented design, $lx_{\left(i\right)}$ and $ly_{\left(i\right)}$ are both equal and vary with the fractal stages, unlike *lz*, which remains constant throughout the different stages. FR4 of thickness *dz* (1 mm) is fixed in its place and doesn't allow any movement of the fluid through it. Due to its fixed nature, $Cz_{eq}^{\left[i\right]}$ doesn't contribute to the detection of moisture content.

The transformer oil is flowing between the two cells, acting as a dielectric medium of a capacitor. $Cx_{eq}^{\left[i\right]}$ and $Cy_{eq}^{\left[i\right]}$ changes with the change in dielectric permittivity of a transformer oil. The dielectric constant of water ($\varepsilon_{water}$=76.546) [29] is much greater than of transformer oil ($\varepsilon_{oil}$=2.1265) [30], which changes the equivalent capacitance even with the slight addition of moisture in oil. Another parameter named ESR, which also depends upon the resistivity of the medium, is measured to increase the accuracy of the proposed design. The extreme difference between the resistivities (at 30 °C) of water ($18.11\times 10^{4}\;\Omega .m$) [31] and oil ($8.83\times 10^{13}\;\Omega .m$) [32] changes the ESR considerably as well.

### 2.3 Mathematical modeling

In the proposed fractal-based state-of-the-art capacitor design, multiple capacitors are formed in all possible directions, i.e., x-axis, y-axis and z-axis. The number of capacitors in a particular direction depends upon the number of fractal stages. These capacitors are arranged in matrices as shown in equation (1) and (4), having dimensions of $n_{\left(i\right)}\times (n_{\left(i\right)}-1)$, $(n_{\left(i\right)}-1)\times n_{\left(i\right)}$, and $n_{\left(i\right)}\times n_{\left(i\right)}$ for $Cx_{eq}^{\left[i\right]}$, $Cy_{eq}^{\left[i\right]}$, and $Cz_{eq}^{\left[i\right]}$, respectively. The fractal stage may range from 0 to ∞.

(1) $$Cx_{eq}^{\left[i\right]}=\left[\begin{array}{cccc} Cx_{1\times 1} & Cx_{1\times 2} & \ldots & Cx_{1\times n_{\left(i\right)}-1}\\ Cx_{2\times 1} & Cx_{2\times 2} & \ldots & Cx_{2\times n_{\left(i\right)}-1}\\ \vdots & \vdots & \vdots & \vdots \\ Cx_{n_{\left(i\right)}\times 1} & Cx_{n_{\left(i\right)}\times 2} & \ldots & Cx_{n_{\left(i\right)}\times n_{\left(i\right)}-1} \end{array}\right]$$

Equation (2) represents the similarity in dimensions of $Cx_{eq}^{\left[i\right]}$ and $Cy_{eq}^{\left[i\right]}$ due to the unchanged medium and distances for capacitors along the x-axis and y-axis. Overall capacitance is measured using the equation (3).

(2) $$Cy_{eq}^{\left[i\right]}=Cx_{eq}^{{\left[i\right]}^{T}}$$

(3) $$C_{eq}^{\left[i\right]}=Cx_{eq}^{\left[i\right]}+Cy_{eq}^{\left[i\right]}+Cz_{eq}^{\left[i\right]}$$

(4) $$Cz_{eq}^{\left[i\right]}=\left[\begin{array}{cccc} Cz_{1\times 1} & Cz_{1\times 2} & \ldots & Cz_{1\times n_{\left(i\right)}}\\ Cz_{2\times 1} & Cz_{2\times 2} & \ldots & Cz_{2\times n_{\left(i\right)}}\\ \vdots & \vdots & \vdots & \vdots \\ Cz_{n_{\left(i\right)}\times 1} & Cz_{n_{\left(i\right)}\times 2} & \ldots & Cz_{n_{\left(i\right)}\times n_{\left(i\right)}} \end{array}\right]$$

In a generalized form, $Cx_{eq}^{\left[i\right]}$, $Cy_{eq}^{\left[i\right]}$, and $Cz_{eq}^{\left[i\right]}$ are represented in equation (5) and (6). The equality of $Cx_{eq}^{\left[i\right]}$ and $Cy_{eq}^{\left[i\right]}$ is due to the same number of capacitors having similar surface area, dielectric medium and distance between two cells. After implementing the conditions, the $C_{eq}^{\left[i\right]}$ is given in equation (7).

(5) $$Cx_{eq}^{\left[i\right]}=Cy_{eq}^{\left[i\right]}=n_{\left(i\right)}\times (n_{\left(i\right)}-1)\times \frac{(lx_{\left(i\right)}\times lz_{\left(i\right)})\varepsilon }{d_{\left(i\right)}}$$

(6) $$Cz_{eq}^{\left[i\right]}=n_{\left(i\right)}^{2}\times \frac{(lx_{\left(i\right)}\times ly_{\left(i\right)})\varepsilon }{dz}$$

Here,

$$lx_{\left(i\right)}=ly_{\left(i\right)}=l_{\left(i\right)}$$

$$lz_{\left(i\right)}=lz$$

$$d_{\left(i\right)}=d$$

(7) $$C_{eq}^{\left[i\right]}=\left(2n_{\left(i\right)}(n_{\left(i\right)}-1)\frac{l_{\left(i\right)}lz\varepsilon_{0}\varepsilon_{m}}{d}\right)+\left(n_{\left(i\right)}^{2}\frac{l_{\left(i\right)}^{2}\varepsilon_{0}\varepsilon_{s}}{dz}\right)$$

Where,

$$lz=lz^{\prime }-dz$$

$$l_{\left(i\right)}\approx 2\pi (a_{\left(i\right)}+b_{\left(i\right)})\times \left(\frac{3{\left(a_{\left(i\right)}-b_{\left(i\right)}\right)}^{2}}{{\left(a_{\left(i\right)}+b_{\left(i\right)}\right)}^{2}\left(\sqrt{-\frac{3{\left(a_{\left(i\right)}-b_{\left(i\right)}\right)}^{2}}{{\left(a_{\left(i\right)}+b_{\left(i\right)}\right)}^{2}}+4}+10\right)}+1\right)$$

$$a_{\left(i\right)}=\frac{L_{\left(i\right)}^{\prime }}{16}\;and\;b_{\left(i\right)}=\frac{L_{\left(i\right)}^{\prime }}{8}$$

$$L_{\left(i\right)}^{\prime }=\frac{L_{initial}^{\prime }-(r-1)d}{r}$$

$$n_{\left(i\right)}=r=2^{i}$$

The dependence of ESR on the dielectric constant and resistivity of the medium is evident in equation (8) [33].

(8) $$ESR=\frac{\varepsilon_{m}\times \rho_{m}}{C_{eq}^{\left[i\right]}}$$

The $\varepsilon_{oil}$, $\rho_{oil}$, $\varepsilon_{water}$, and $\rho_{water}$ changes with the temperature and hence varying the $\varepsilon_{m}$ [34] and $\rho_{m}$ [32]. The dielectric constant and resistivity of mixed medium depends upon the PPM and temperature, as given in Table 2.

**Table 2:** Dielectric constant and resistivity.

Output	Input	Function
*ε*_*m*_	PPM, T	$\varepsilon_{oil}+\frac{3\varepsilon_{oil}\times \frac{PPM}{10^{6}}}{\frac{\varepsilon_{water}-2\varepsilon_{oil}}{\varepsilon_{water}-\varepsilon_{oil}}-\frac{PPM}{10^{6}}}$
*ρ*_*m*_	PPM, T	*u*(*X*)*ρ*_*oil*_ + *v*(*Y*)*ρ*_*water*_

The condition which limits the fractal stage to reach ∞ is given in equation (9).

(9) $$L_{initial}^{\prime }>(r-1)d$$

The volume of an overall capacitor can be calculated using given equation (10).

(10) $$V_{\left(i\right)}={\left(L_{initial}^{\prime }-2(r-1)d\right)}^{2}\times lz$$

## 3 Substages and switching mechanism

In the ongoing section, the role of the switching mechanism is discussed. As a result of switching, further substages of the fractal stage are formed to make the FSCS reconfigurable. The concept of substages is explained in detail, along with the equivalent circuit of switching.

### 3.1 Substages

**Figure 8:**
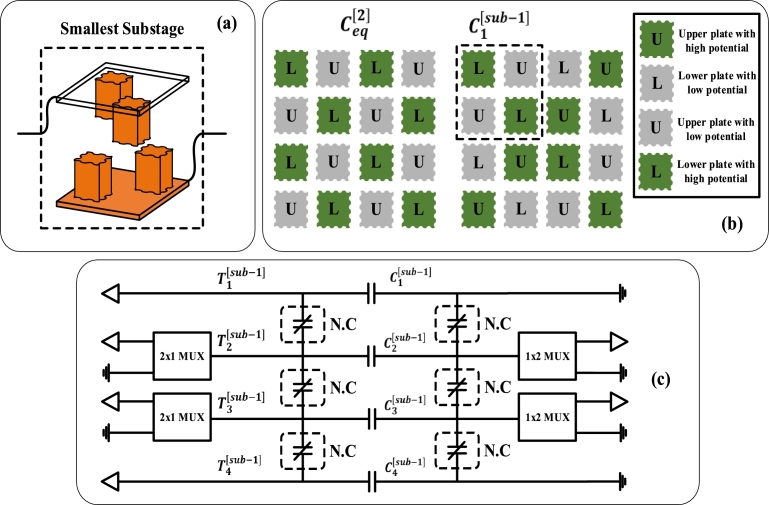
(a) The smallest substage possible of any fractal stage (b) The specific pattern of upper plates with high, low potential and lower plates with high, low potential is selected to switch between substages of a particular fractal stage (c) The conceptual circuit diagram of switching mechanism with 2x1 MUX, 1x2 MUX and contactors.

The substages are further stages in a particular fractal stage. For instance, a fractal stage 2 has two substages, as shown in Fig. 8. The substage 0 and $i^{th}$ fractal stage are equal and doesn't have any distinct properties. The number of cells along the x or y-axis remains the same for both $i^{th}$ fractal stage and substage 0, as can be seen from equation (11). Meanwhile, for substages greater than 0 or less than *i*, the number of cells and, hence, capacitance are modified. Similarly, in the case of the dielectric constant of a mixed medium, it remains the same for $i^{th}$ fractal stage and substage 0 and varies otherwise, which is evident from equation (12).

(11) $$n_{\left(i\right)}^{\left[sub-j\right]}=\left\{ \begin{array}{cc} n_{\left(i\right)}, & j=0\\ \frac{n_{\left(i\right)}^{\left[sub-(j-1)\right]}}{2}, & 0<j<i \end{array}\right.$$

(12) $$\varepsilon_{m}^{\left[sub-j\right]}=\left\{ \begin{array}{cc} \varepsilon_{m}, & j=0\\ 4^{j}\times \varepsilon_{m}, & 0<j<i \end{array}\right.$$

It is worth noting that the smallest substage of any fractal stage is shown in Fig. 8 (a), which contains 2 cells along the x and y-axis. As can be seen in Fig. 8 (b), $C_{eq}^{\left[2\right]}$ consist of multiple cells, with each alternate cell having opposite polarity as to the nearby cell. Whereas, $C_{1}^{\left[sub-1\right]}$ have different patterns of polarities according to the switching mechanism described in the next section.

### 3.2 Switching mechanism

Each fractal stage has *j* substages, and each stage contains the smallest substage shown in Fig. 8 (a). The two terminals connected with the upper and lower plate of the smallest substage are shown. In the case of fractal stage 2, while switching from substage 0 to 1, the polarities of cells change such that there is no capacitance formation with the nearby substage, as shown in Fig. 8 (b). It is also evident from the figure shown that only 2 of 4 substages change the polarity. This trend remains constant as fractal stages increase.

As shown in Fig. 8 (c), there is a connection of MUX with only 2 substages instead of 4 to change their polarity, which is described above. Also, a number of normally closed contactors are included in the equivalent circuit. For substage 0, the contactors remain closed, while for other substages, these get open to calculate the equivalent capacitance of each substage separately.

## 4 Fabrication and experimental setup

**Figure 9:**
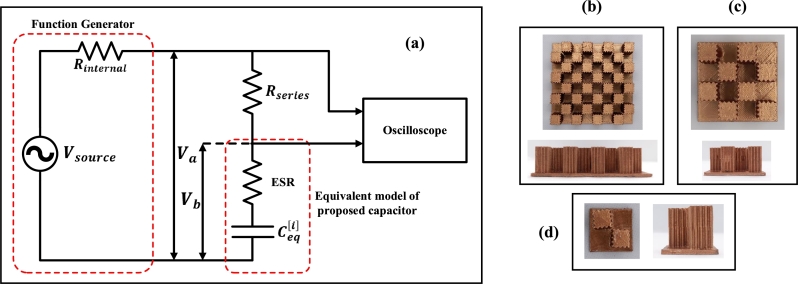
(a) The circuit diagram of an experimental setup containing a function generator to provide voltage signal of a selected frequency, series resistor, capacitor under test and oscilloscope (b) Top and Side view of fractal stage 3's substage 0 (c) substage 1 and (d) substage 2.

To test the proposed FSCS, it is fabricated and integrated with other electrical equipment to measure the capacitance at different moisture content. The fabricated sensor is of fractal stage 3 with $L_{initial}^{\prime }=8cm$, $d_{\left(i\right)}=d=0.1cm$, dz=0.1cm, $l_{z}=1.9cm$, $\epsilon_{s}=4.4$. The equivalent circuit of the experimental setup is shown in Fig. 9 (a), where a resistor is connected in series with the proposed FSCS. A function generator with its internal resistance is connected to the circuit to generate a signal at a specific frequency. The two-channel oscilloscope is attached to the output of a circuit. One and two channels are used to measure $V_{a}$ and $V_{b}$, respectively. By measuring the $V_{a}$ and $V_{b}$, the capacitance and ESR of FSCS can be calculated using equation (13) and (14), respectively.

(13) $$C_{eq}^{\left[i\right]}=\frac{1}{2\pi f\left(\frac{V_{b}^{\left[i\right]}cos(\phi )\times R_{series}}{V_{R_{series}}^{\left[i\right]}}\right)}$$

(14) $$ESR^{\left[i\right]}=\frac{V_{b}^{\left[i\right]}sin(\phi )\times R_{series}}{V_{R_{series}}^{\left[i\right]}}$$

The fabricated substages 0, 1 and 2 of Fractal Stage 3 FSCS are shown in Fig. 9 (b), (c), and (d), respectively. For the fabrication, a 3D model of FSCS is created and printed with the copper (Cu) filament. The electrodes are printed separately and then assembled with an FR4 substrate for support in between. Once an FSCS is assembled, it is placed in a specially designed enclosure which has one inlet and outlet. A control unit is attached to the FSCS to measure the capacitance, ESR and temperature of the medium. All of the fabricated substages of Fractal Stage 3 are tested to compare the measured and calculated values of capacitance and ESR.

## 5 Results and discussions

The simulation and calculations are done for the FSCS design. The promising correlation between simulation and calculation is recorded, which shows the accuracy of mathematical modeling. The comparison of designs, the effect of moisture and temperature, and experimental results are shown and discussed in the upcoming subsections.

### 5.1 Comparison of two designs

**Figure 10:**
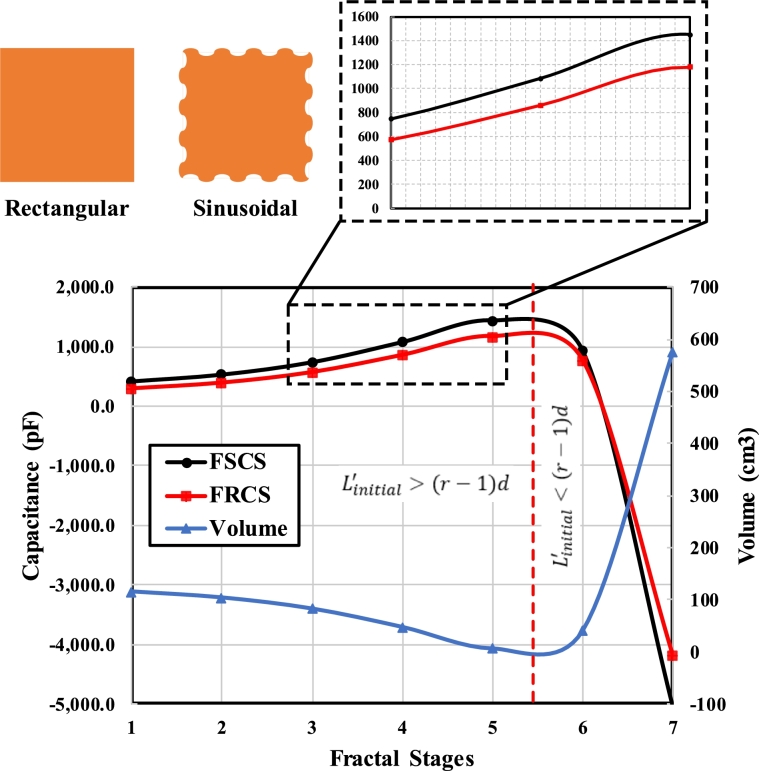
Comparison of the equivalent capacitances of Rectangular and Sinusoidal-shaped capacitors. The total volume of used material is also plotted against fractal stages.

A fractal-based Rectangular shaped Capacitive Sensor (FRCS) is compared with the FSCS. A 29.24%, 26.39%, 23.19%, 20.91% and 18.62% capacitance is increased by changing FRCS with the FSCS for fractal stages 1, 2, 3, 4 and 5, respectively. A comparison is made to see the improvement in the contact area when using FSCS instead of FRSC. The rise of capacitance shows an increase in the contact area of FSCS compared to the FRCS, thus improving sensitivity. It can be seen in Fig. 10 that the response shows the decay in the volume of used material while increasing the overall capacitance. It also increases the contact area of capacitor plates with the medium under inspection, which increases the contamination sensing capability of a proposed sensor.

The rise of capacitance and decay of volume is valid until the condition shown in (9) holds true. Once the condition doesn't hold, the volume of material shoots upward and capacitance downward.

### 5.2 Capacitance across different substages

**Figure 11:**
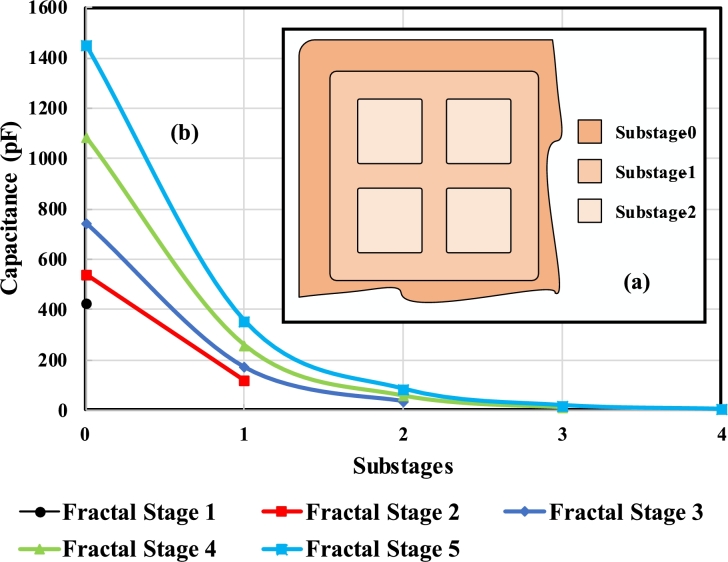
(a) Concept of substages for a given stage (b) Change in equivalent capacitance of different fractal stages at various substages.

The substage of each fractal stage has a different value of capacitance as compared to the other. The concept of substages is again demonstrated using a graphical depiction and a diagram, as shown in Fig. 11. In Fig. 11 (a), a portion of Fractal Stage 3, along with its substages, is given. There are three different colours which are indicating substages 0, 1 and 2. A detailed explanation of this concept is discussed above in section 3.

In Fig. 11 (b), a trend of equivalent capacitance against different substages of various fractal stages is depicted. For the fractal stage 0, there is only one substage, i.e. substage 0. Each fractal stage has a different number of substages, as described above. The trend of decrease in equivalent capacitance due to the increase in substage is clearly visible. This is because of the lesser number of cells included in the higher substage capacitor design and, hence, less overall equivalent capacitance. Hence, even though the sensitivity of the higher substage is lesser, due to the higher ratio of transformer oil to the moisture content in higher substages, small contamination can also be detected easily. As the contact area of impurity with the electrodes is increased in case of higher substage, the sensor is operated in large contact area mode.

### 5.3 Effect of moisture content on capacitance and ESR

As described above, a slight addition of moisture content in the transformer oil medium has a predominant effect on the mixture's overall dielectric constant. The role of the fractal stage in determining the sensor's sensitivity is described by measuring the equivalent capacitance upon the addition of moisture content.

**Figure 12:**
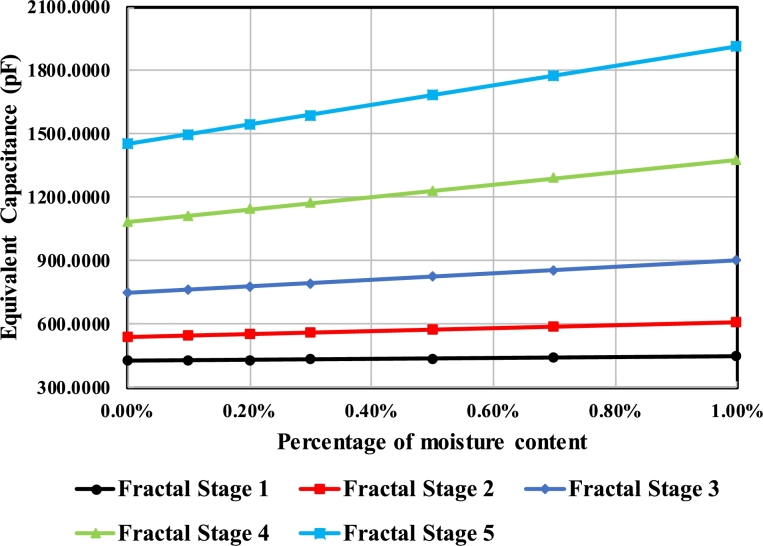
Effect of moisture content (%age) on the equivalent capacitance of each fractal stage.

The effect of moisture content on the equivalent capacitance is shown in Fig. 12. The sensing capability of the FSCS increases as the contact area between Capacitor plates and dielectric medium increases. It is quite evident from Fig. 12 that higher fractal stages have higher sensing capabilities as compared to the lower ones. A change of 5.34%, 11.5%, 17.7%, 21.39% and 24.06% is seen when capacitance is measured on 0% and 1% moisture content for five fractal stages.

**Figure 13:**
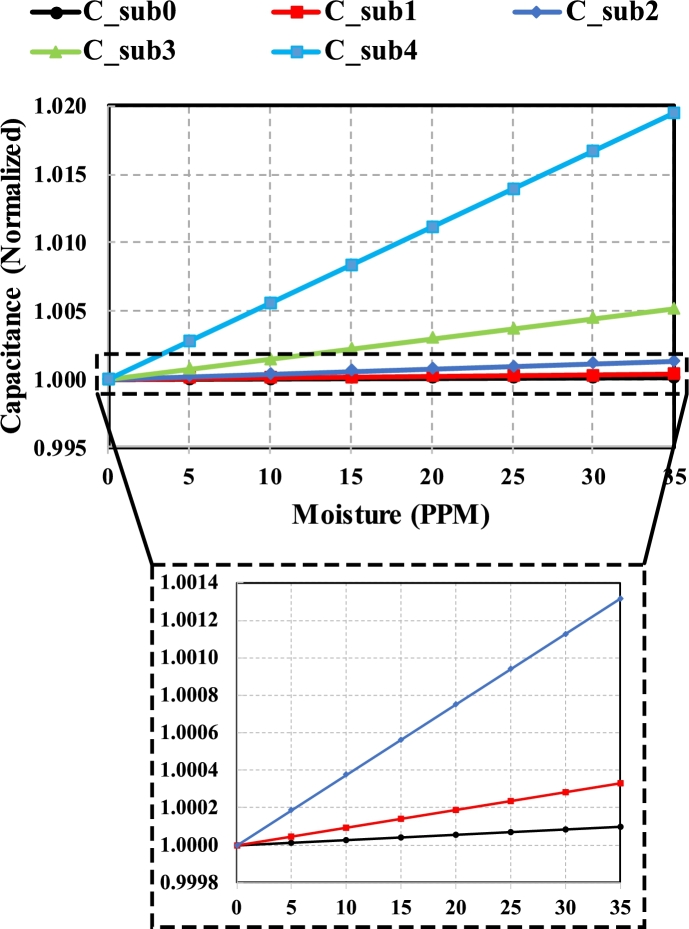
Effect of moisture (PPM) on a Normalized Capacitance of 5 substages of fractal stage 5.

In the ongoing discussion, the equivalent capacitance of each substage of fractal stage 5 is calculated based on different quantities of moisture varying from 0 to 35PPM. The addition of moisture increases the overall dielectric constant of fluid. Due to the change in the dielectric medium, the equivalent capacitance rises, as can be seen in Fig. 13.

The normalized capacitance of substage 0, 1, 2, 3, and 4 rises from 1 to $9.8\times 10^{-5},3.31\times 10^{-4},1.315\times 10^{-3},5.172\times 10^{-3}$, and $1.951\times 10^{-2}$ as moisture content changes from 0 to 35PPM, respectively. The greater change in higher substages is due to the higher ratio of transformer oil to moisture content (large contact area mode). The dielectric constant of mixed medium for substages 0, 1, 2, 3, and 4 increases from 2.09872 to 2.0989, 2.0994, 2.1018, 2.111, 2.148. In substage 4, a change of 2.29% in dielectric constant is seen as compared to the $8.5\times 10^{-3}$% change for substage 0. This considerable change is useful in detecting less amount of contamination easily.

Hence, a lower substage is useful in achieving higher sensitivity, as shown in Table 3. Once the moisture content is detected in the lower substage, the sensor is reconfigured into the higher substage by means of switching. The benefit of a higher substage is the higher ratio of transformer oil to moisture content, which allows for accurate detection and hence avoids any false alarms. The higher ratio of the two mediums increases the contact area of impurity with the electrodes of the capacitor.

**Table 3:** Sensitivity (fF/5PPM).

		Fractal Stages				
		1	2	3	4	5
	0	1.0463	3.0594	6.7678	12.9131	20.1179
	1	-	1.7262	4.9094	10.2	16.4769
Substages	2	-	-	3.2730	8.7428	15.3784
	3	-	-	-	5.8286	13.1815
	4	-	-	-	-	8.7877

**Figure 14:**
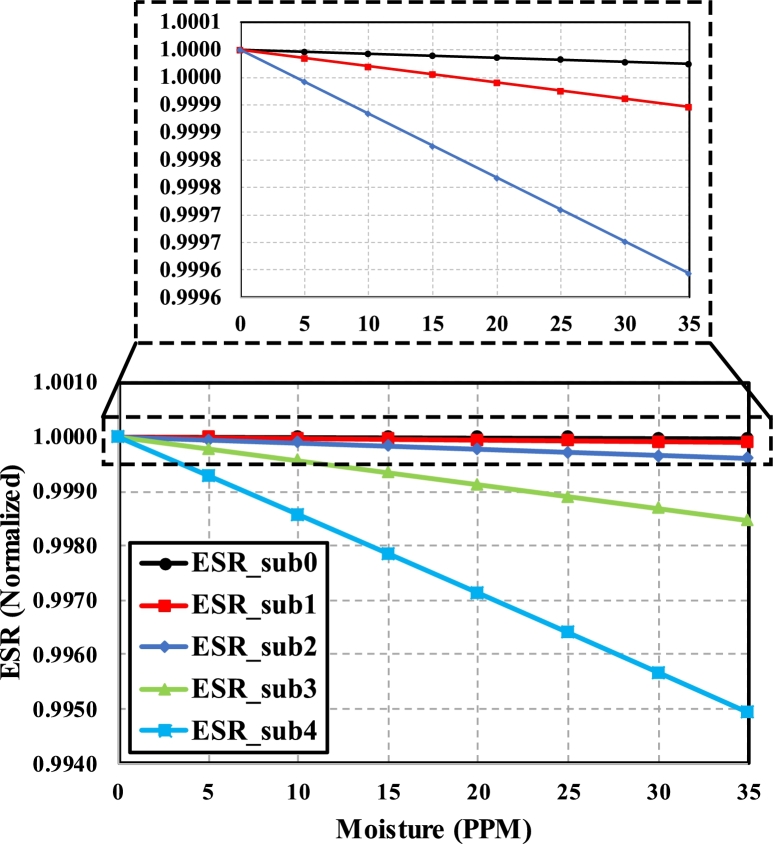
Change in normalized ESR of various substages as moisture content changes from 0-35PPM.

Another parameter, ESR, is calculated along with the equivalent capacitance to reduce the reading error and false alarm situation. In contrast to the capacitance, ESR decreases as moisture content increases, as shown in Fig. 14. According to (8), ESR is directly proportional to the product of $\varepsilon_{m}$ and $\rho_{m}$. The addition of moisture content causes the resistivity of mixed medium to decrease considerably. The decrement is considerable as compared to the slight increment in dielectric constant, due to which an overall decreasing trend is shown in Fig. 14. Similar to the capacitance, normalized ESR also shows considerable change at higher substages.

The sensitivity of each substage of fractal stages is shown in Table 3 and measured in fF/5PPM. The sensitivity of substage 0 increases as the fractal stage gets higher. For the higher substages of any particular fractal stage, sensitivity decreases due to the decrease in equivalent capacitance. There is a tradeoff between increased contact area with the impurity and higher sensitivity when changing between the different substages of any fractal stage. However, due to the reconfigurability of an FSCS, large contact area mode and higher sensitivity mode can both be activated at different times.

### 5.4 Dependency of oil and water on temperature

Generally, a single value for dielectric constant and resistivity is taken at a certain temperature to simplify the problem. In practice, the effect of temperature on the properties of the dielectric cannot be ignored. To achieve maximum accuracy while sensing, a temperature sensor takes readings in live time to account for the changes in dielectric constant and resistivity values.

**Figure 15:**
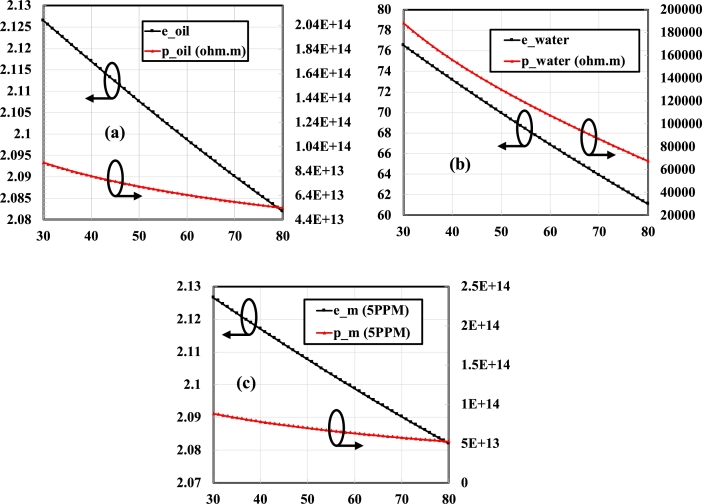
Effect of temperature on (a) transformer oil permittivity, resistivity, (b) water permittivity, resistivity and (c) mixture permittivity and resistivity Ω.*m*.

An upward trend for $\varepsilon_{oil}$, $\varepsilon_{water}$, $\varepsilon_{m}$ (5PPM), and a downward trend for $\rho_{oil}$, $\rho_{water}$, $\rho_{m}$ (5PPM) with the rise in temperature is visible in Fig. 15 (a, b, c).

The value of $\varepsilon_{oil}$, $\rho_{oil}$, $\varepsilon_{water}$, $\rho_{water}$, $\varepsilon_{m}$ (5PPM), and $\rho_{m}$ (5PPM) changes from 2.14 to 2.06, 1.28$\times 10^{14}(\Omega .m)$ to $4.45\times 10^{13}(\Omega .m)$, 83.776 to 55.705, $2.8148\times 10^{5}(\Omega .m)$ to $3.138\times 10^{4}(\Omega .m)$, 2.1462 to 2.0672 and $2.2629\times 10^{11}(\Omega .m)$ to $7.8541\times 10^{10}(\Omega .m)$ at 10 °C to 100 °C.

The trend of dielectric constant and resistivity of mixed medium with the change in temperature is shown only for the single moisture content quantity, i.e. 5PPM. For higher quantities, an overall upward and downward trend remained the same for dielectric constant and resistivity, respectively. But at a certain temperature, an increase in the dielectric constant and a decrease in the resistivity from the lower quantity is seen.

### 5.5 Experimental results

The required experimental setup to test the proposed sensor is explained in section 4. The fabricated sensors of fractal stage 3 having substage 0, 1, and 2 are also shown in the same section. The upper and lower plates of the sensor are fabricated precisely and joined using FR4 substrate. Other details of the enclosure and control unit are given in section 4.

**Figure 16:**
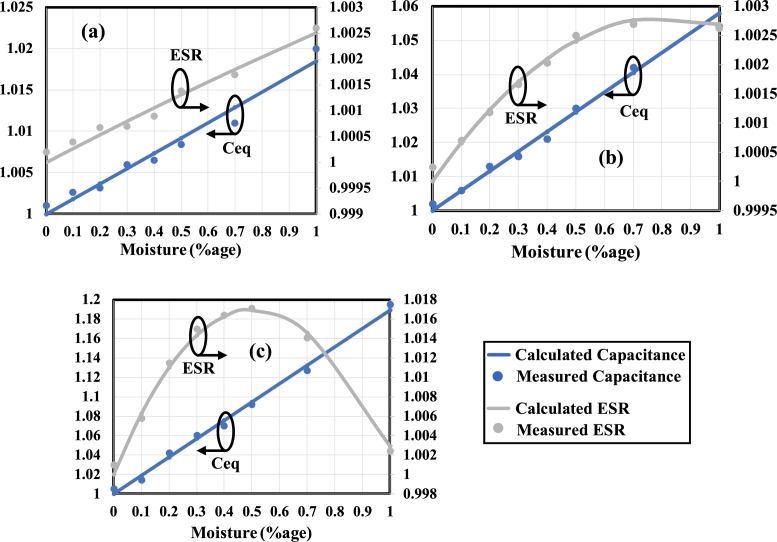
Comparison of calculated and measured values of normalized Capacitance and ESR of fractal stage 3 at (a) substage 0, (b) substage 1, and (c) substage 2.

The experimental results of the capacitance and ESR of substages 0, 1 and 2 of Fractal Stage 3 are compared with the calculated results as shown in Fig. 16. The actual transformer oil sample is provided by the High Voltage & Short Circuit Lab, NTDC. The FSCS is tested at a moisture content between 0.1% to 1%. The measurements are first taken using the experimental setup containing a function generator, and results are shown in Fig. 16 (a, b, c). There is a slight variation in calculated and measured values due to the reading error, which reduces as a higher substage is tested. At higher substages, the difference between values of capacitance and ESR on the addition of a certain moisture content (%age) becomes higher and can easily be detectable.

To crosscheck the values, the same setup is connected with the UNI-T UT612 LCR Meter in order to measure the capacitance and ESR. While doing so, the function generator is disconnected, and the FSCS terminals are connected directly to the meter. The control unit still controls the inlet and outlet of an enclosure. All the measurements are crosschecked using this method. It is found that the measurements taken from both methods are close to each other. However, the fluctuation of the values is seen while taking the reading through the meter. It is recommended that the experimental setup explained in section 4 be used to get stable values of capacitance and ESR.

### 5.6 Comparison with the existing sensors

This problem has been under research for a long time, and there are many methods, ranging from the use of optical fibre to acoustics, to detect the moisture content in insulation oil. However, the research done in this paper uses the capacitive sensor. Research has also been done using this technique, but the solution provided in this paper is unique as it introduces the concept of switching to change the mode of operation. In [35], a sensitive thin film-based capacitive sensor is used for moisture measurement in oil. Two parameters were measured in this experiment: capacitance and dissipation factor. The variation in capacitance with respect to temperature is also measured. In [33], a diagnosis system for transformer oil is designed based on the frequency response analysis of the capacitive sensor. The temperature is kept steady at 70 ^∘^C, without considering any changes. Table 4 provides a comparison between the previously proposed sensors and the solution presented in this paper.

**Table 4:** Comparison with the existing sensors.

Reference	Sensing method	Temperature variation	Parameters measured	Contamination	Range	Reconfigurability
[35]	Impedance Analyzer	Yes	Capacitance, Dissipation factor	Moisture	pF	No
[33]	Frequency Response Analysis	No	Gain	Moisture	fF	No
[26]	Capacitance	No	Capacitance	Moisture and 2-Furfuraldehyde	fF	No
[36]	Capacitance	No	Capacitance	Moisture	nF	No
This paper	Capacitance	Yes	Capacitance and ESR	Moisture	fF	Yes

## 6 Conclusion

In this paper, a novel FSCS design is proposed for the detection and continuous monitoring of moisture content in transformer oil. The design comprised a Sinusoidal-shaped side instead of a Rectangular-shaped side to increase the contact area between capacitor plates and dielectric medium by 17.3%. The effect of temperature on the dielectric constant and resistivity of transformer oil and water was taken into account to increase detection accuracy. Furthermore, a switching mechanism was integrated with the FSCS to enhance its contact area with the impurity and sensitivity at different times, making it reconfigurable. The contact area with the impurity was increased by increasing the substage, and sensitivity was increased by decreasing the substage of a particular fractal stage. It was seen that at higher substages, lower equivalent capacitance is achieved compared to the lower substages, which is a cause of low sensitivity. Approximately 75.73% decrease in the capacitance was seen while switching from substage 0 to 1 of Fractal Stage 5. In substage 4 of Fractal Stage 5, a change of 2.29% in dielectric constant was seen while increasing the moisture content from 0 to 35PPM due to the activation of large contact area mode. On the other hand, a change of only $8.5\times 10^{-3}$% in dielectric constant is recorded for substage 0 of the same fractal stage 5. To conclude, a minimum detection limit of 5PPM is achieved with the sensor. The sensor reaches a maximum sensitivity of 20.1179 fF per 5PPM using a Fractal stage 5 design. For future research, the sensor's ability to detect moisture content when it is exposed to different contaminants, such as metal and dust particles, needs to be tested.

## CRediT authorship contribution statement

**Muhammad Fahad Naveed:** Writing – original draft, Validation, Methodology, Formal analysis, Conceptualization. **Akasha Amaar:** Writing – review & editing, Software. **Saad Saleem Khan:** Project administration. **Muhammad Omar:** Supervision. **Stephen Larkin:** Funding acquisition.

## Declaration of Competing Interest

The authors declare the following financial interests/personal relationships which may be considered as potential competing interests: Muhammad Fahad Naveed reports financial support and article publishing charges were provided by Africa New Energies Ltd (ANE-088). Muhammad Omar reports a relationship with Africa New Energies Ltd that includes: funding grants. If there are other authors, they declare that they have no known competing financial interests or personal relationships that could have appeared to influence the work reported in this paper.

## Data Availability

Data included in article/supp. material/referenced in article.
